# Cancer-associated fibroblasts: from basic science to anticancer therapy

**DOI:** 10.1038/s12276-023-01013-0

**Published:** 2023-07-03

**Authors:** Dakai Yang, Jing Liu, Hui Qian, Qin Zhuang

**Affiliations:** 1 Department of General Practice, Affiliated Hospital of Jiangsu University, Zhenjiang, People’s Republic of China; 2 Department of Laboratory Medicine, School of Medicine, Jiangsu University, Zhenjiang, People’s Republic of China; 3 Microbiology and Immunity Department, Shanghai, People’s Republic of China; 4 Collaborative Innovation Center for Biomedicines, Shanghai University of Medicine & Health Sciences, Shanghai, People’s Republic of China

**Keywords:** Cancer microenvironment, Oncogenesis

## Abstract

Cancer-associated fibroblasts (CAFs), as a central component of the tumor microenvironment in primary and metastatic tumors, profoundly influence the behavior of cancer cells and are involved in cancer progression through extensive interactions with cancer cells and other stromal cells. Furthermore, the innate versatility and plasticity of CAFs allow their education by cancer cells, resulting in dynamic alterations in stromal fibroblast populations in a context-dependent manner, which highlights the importance of precise assessment of CAF phenotypical and functional heterogeneity. In this review, we summarize the proposed origins and heterogeneity of CAFs as well as the molecular mechanisms regulating the diversity of CAF subpopulations. We also discuss current strategies to selectively target tumor-promoting CAFs, providing insights and perspectives for future research and clinical studies involving stromal targeting.

## Cancer: Reprogramming cells that support tumors

Tumors reprogram nearby wound-healing cells into cancer-associated fibroblasts (CAFs) to support their metabolism, escape the immune response and develop resistance to chemotherapy; targeting CAFs may provide therapeutic opportunities. CAFs are very diverse, and their origins and specific roles are not well understood. New genetic tools allow precise profiling of CAFs and their functions, and Dakai Yang at Jiangsu University in Zhenjiang, China, and co-workers have reviewed CAF diversity and the mechanisms by which they are generated. Although most CAFs support tumors, some CAFs fight tumors, and they can potentially be converted from one form to another. Improving our understanding of the variety of CAFs, their functions, and how they interact with tumor cells may help in identifying tumor-suppressing CAFs and in developing precision medicine treatments for various types of cancer.

## Introduction

The concept of the tumor microenvironment (TME), although first proposed by Stephen Paget in the “seed and soil theory” in 1889, has been widely appreciated only in recent years based on mounting evidence showing that the heterotypic signaling among the diverse cell types within a tumor cooperatively creates a supportive niche that favors cancer cell survival, outgrowth and escape from immunosurveillance^1^. Thus, the behavior of cancers depends not only on cancer cell-autonomous defects but also on cancer cell-extrinsic factors, in particular, the intricate tumor microenvironment (TME). The TME, as a dynamic niche composed of a set of cellular and molecular components, closely interacts with cancer cells to meet their nutrient and metabolic demands^2^. Diverse cell types contribute to TME formation, including immune cells, cancer-associated fibroblasts (CAFs) and even normal epithelial cells, which together are termed stromal cells^3^. Soluble factors, such as growth factors and cytokines, as well as capillaries and the extracellular matrix (ECM) surrounding cancer cells, together constitute the complex network of tumor stromal signaling mediated by both tumor and stromal cells^3^.

CAFs, also known as activated fibroblasts, are central to the reactive stroma within the TME, as they not only interact extensively with cancer cells via secreted molecules or cell‒cell adhesion but also indirectly influence cancer cells via ECM remodeling and immune cell infiltration^4^. While there is an overwhelming abundance of studies that support a tumor-promoting function for CAFs, some studies have noted that CAFs may restrain cancer progression as a host defense mechanism against neoplasia^5,6^. This contradiction can be explained by the high heterogeneity and plasticity of CAFs, which are possibly dependent on CAF precursor origin, cancer type and tumor progression stage. Indeed, distinct CAF subtypes have been identified with specific molecular markers, such as myofibroblast-like CAFs (myCAFs)^7^, inflammatory CAFs (iCAFs)^7^ and antigen-presenting CAFs (ApCAFs)^8^, which perform different and, in some cases, even contradictory roles in tumorigenesis. Moreover, recent studies using single-cell RNA-sequencing and proteomic technology have further dissected CAF subpopulations based on their distinct transcriptional profiles and demonstrated dynamic heterogeneous modifications of stromal myofibroblast populations in a context-dependent manner^2,9,10^. The variations in stromal composition not only shape the intratumoral architecture but also contribute to functional changes in tumor cell behavior, highlighting the importance of precise assessment of CAFs when considering treatment options. In this review, the proposed origins and heterogeneity of CAFs are summarized with a special focus on the therapeutic potential of CAFs. We also present recent advancements in specific targeting of protumorigenic CAFs or interference with their activity as potential strategies in anticancer therapy.

## Origins and Heterogeneity of CAFs

### Fibroblasts and acute wound healing

Initially identified in a wound healing response, fibroblasts play a central role in tissue repair, in which quiescent local tissue fibroblasts respond to tissue injury and become reversibly activated to initiate regenerative repair^11^. Activated fibroblasts, also termed myofibroblasts due to their high expression of α‑smooth muscle actin (α-SMA), acquire the capabilities of enhanced contractility, ECM production and inflammatory mediator secretion that together initiate wound healing responses, such as closure of the wound and production of connective tissue^12^. Interestingly, myofibroblasts also contribute to ECM turnover at the late stage of tissue repair with the synthesis of ECM-degrading proteases, such as matrix metalloproteinases (MMPs) and urokinase-type plasminogen activator (uPA), thus facilitating the restoration of the normal tissue architecture without scarring^13^. Once the repair process is complete, these transiently activated fibroblasts undergo either apoptosis or reprogramming to the resting state^14^.

### Fibroblasts and chronic wound healing/cancer

Unlike the acute wound healing response, which is a natural physiological reaction to acute tissue injury, chronic or repetitive injury such as toxic, metabolic, or infectious insult often results in continuous activation of fibroblasts and excessive ECM component deposition, ultimately leading to pathological tissue fibrosis with impaired organ function^15^. One of the key mechanisms underlying tissue repair versus irreversible fibrosis is that in acute wound repair, fibroblasts are transiently activated, while during repetitive damage, fibroblasts become resistant to apoptosis or have a limited ability to reacquire a quiescent phenotype^14^.

Cancer, especially solid tumors, has long been considered a nonhealing wound^16^ and shares many features with tissue fibrosis, such as activated fibroblasts and increased stiffness of the ECM^17^. Fibroblasts at the site of a tumor, specifically referred to as CAFs, remain perpetually activated with a high capacity for ECM synthesis and microenvironmental remodeling, leading to stromal desmoplasia, a phenomenon characterized by increased deposition of ECM components in tumors. In this regard, CAFs share many basic characteristics, such as a secretory phenotype and capacity to synthesize ECM components, with fibroblasts found in nonmalignant tissue fibrosis. Therefore, the classic markers found to be expressed in fibroblasts, including α-SMA, vimentin, desmin, fibroblast-specific protein 1 (FSP1; also known as S100A4) and fibroblast activation protein (FAP), have been conventionally used to distinguish CAFs in recent years^3,18^. Notably, CAFs, while remodeling the TME and influencing cancer cell behavior, are also directly or indirectly reprogrammed by cancer cells and other stromal cells, ultimately displaying distinct epigenetic and transcriptional profiles correlated with their robust proliferative and invasive properties^19^. Thus, a set of surface markers, such as platelet-derived growth factor receptor-α/β (PDGFRα)/PDGFRβ^20^, discoidin domain-containing receptor 2 (DDR2)^21^ and integrin α11β1^22^, have emerged to identify CAFs in the context of a specific TME. Interestingly, it is suggested that the same marker at different expression levels may define the CAF subsets associated with specific stages of cancer development, as loss of caveolin1 (CAV1) is found in metabolically reprogrammed CAFs that promote tumorigenesis^23^, while high CAV1-expressing CAFs contribute to invasion and metastasis in breast cancer^24^. However, none of these markers is exclusively expressed by CAFs; for example, desmin and PDGFRβ are also present in perivascular cells^25^, while FAP is expressed in a subset of CD45^+^ immune cells^26^, suggesting the possibility of diverse cellular origins of CAFs and necessitating in-depth biological deciphering of CAF evolution. Indeed, single-cell RNA-sequencing (scRNA-seq) analysis, which enables profiling of gene expression over the whole transcriptome at single-cell resolution, indicates that no single marker appears capable of discriminating CAFs from all other cell types and or even discriminating CAF subtypes^27–29^. Thus, combinations of two or more biomarkers with high discriminatory capacity are emerging to differentiate and isolate all CAFs across distinct cancer types (Table 1).

**Table 1:** Pan-CAF markers and specific markers for the identification of CAF subtypes.

Fibroblast markers^3,18^	CAF positive markers	CAF negative markers	CAF subtype markers^8^		
			myCAF	iCAF	apCAF
ACTA2	PDGFRα / PDGFRβ^19^	CD45^8^	ACTA2	IL6/IL8	H2-Ab1
VIM	DDR2^20^	CD31^8^	TAGLN	PDGFRA	CD74
COL1	COL1^8^	EPCAM^8^	MMP11	CXCL1/CXCL2/CXCL12; CCL2	SLPI
FSP1 (also known as S100A4)	FAP^8^	PECAM1^18^	HOPX	CFD	SAA3
FAP	PDPN^8^	NG2^19^	POSTN	DPT	
DES	DCN^8^		TPM1/TPM2	LMNA	
	VIM^8^			AGTR1	
				HAS1	

*ACTA2* actin alpha 2, *VIM* vimentin, *COL1* collagen type I, *FSP1* fibroblast-specific protein 1, *FAP* fibroblast activation protein, *DES* desmin, *DDR2* discoidin domain-containing receptor 2, *PDPN* podoplanin, *DCN* decorin, *PECAM1* platelet/endothelial cell adhesion molecule, *EPCAM* epithelial cell adhesion molecule, *TAGLN* transgelin, *MMP11* matrix metallopeptidase 11, *HOPX* homeobox transcription factor, *TPM1/TPM2* tropomyosin 1/2, *CFD* complement factor D, *DPT* dermatopontin, *LMNA* lamin A, *AGTR1* angiotensin II receptor type 1, *CXCL* CXC-chemokine ligand, *Has1* hyaluronan synthase, *H2-Ab1* histocompatibility 2, *class II antigen A* beta 1 gene, *SLPI* Peptidase inhibitor, *Saa3* serum amyloid A3.

### Cellular origins of CAFs

#### CAFs derived from tissue resident fibroblasts or nonfibroblast lineages

**Fig. 1:**
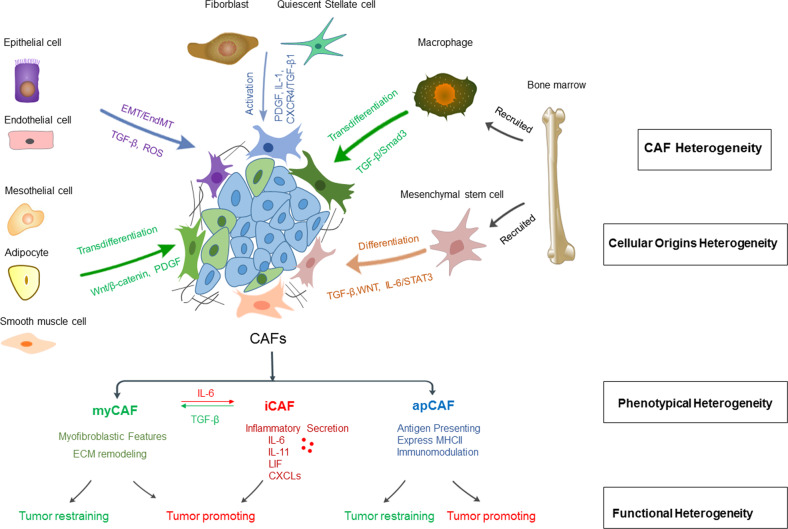
Cancer-associated fibroblasts (CAFs) are a heterogeneous and plastic population within the tumor microenvironment. The heterogeneity of fibroblasts could be attributed to the multiple origins of the precursor cells, the phenotypical diversity of subsets and the distinct function of each subset. Potential cellular sources include local tissue resident stellate cells and normal fibroblasts and nonfibroblast lineage or recruited bone marrow-derived mesenchymal stem cells (MSCs) and macrophages. The main subsets of CAFs include myCAFs, iCAFs and apCAFs, which exhibit different biological features and result in phenotypical diversity and functional heterogeneity in cancer progression. However, the distinct subsets of CAFs are not permanent but interconvertible via manipulation of specific signaling, as shown by the conversion between iCAFs and myCAFs via the TGFβ or IL-6 signaling pathway of CAFs. myCAFs: myofibroblast-like CAFs; iCAFs: inflammatory CAFs; apCAFs: antigen-presenting CAFs.

Although generally termed CAFs to describe all activated fibroblasts in the TME of solid cancers, CAFs are actually highly heterogeneous populations that can originate from disparate precursors (Fig. 1). To date, the precise cellular origins of CAFs remain elusive owing to their substantial heterogeneity and a lack of definitive biomarkers for each subset. The most direct source of CAFs is normal resident fibroblasts or quiescent stellate cells. CAFs in the pancreas and liver are traditionally thought to originate from pancreatic stellate cells (PSCs)^30^ and hepatic stellate cells (HSCs)^31^, respectively, under the influence of tumor-derived stimuli. For example, cancer cell-derived chemokines, cytokines and microRNAs are found to activate PSCs or HSCs, enabling them to gain myofibroblast-like features and transcriptional signatures associated with CAFs in pancreatic ductal adenocarcinoma (PDAC) or hepatocellular carcinoma^32–34^. Activated PSCs and HSCs further maintain their own activity with enhanced synthetic and secretory capacities via autocrine loops and contribute to desmoplasia^35^. Although extensively studied in vitro, the contribution of PSCs to PDAC CAFs in vivo in the context of tumorigenesis remains elusive. In a recent work using a lineage-labeling approach to trace the fate of PSCs in vivo, Helms et al. found that, contrary to expectations, PSCs only give rise to a small minority of CAFs in PDAC^36^, suggesting the existence of diverse CAF progenitors and raising an important question that needs to be addressed regarding the additional cellular origins of PDAC CAFs.

In fact, normal resident fibroblasts, which reside around tumor cells, have been found to be activated via the tumor cell-derived signaling pathway and give rise to a subset of CAFs in PDAC^37^, gastrointestinal cancer^38^ and breast cancer^20^. Furthermore, even local fibroblasts are not homogenous but consist of distinct populations^39^; therefore, current studies attempt to trace specific lineages of fibroblasts and delineate their contribution to stroma formation. In a recent study using lineage tracing and dual recombinase approaches to follow the fate of 2 normal fibroblast populations marked by the expression of Gli1 and Hoxb6, Garcia et al discovered that Gli1, but not Hoxb6, specifically contributes to a portion of PDAC CAFs^40^. Similarly, Kobayashi et al. found a CAF subset marked by melanoma cell adhesion molecule (MCAM) derived predominantly from intestinal pericryptal leptin receptor (Lepr) lineage cells^38^, suggesting that inherent fibroblast heterogeneity may be linked to a specific subpopulation of CAFs.

In addition to the mesenchymal lineage, CAFs have been found to originate from multiple nonfibroblast lineage cells, including epithelial^41^ and endothelial cells^41^, through epithelial/endothelial-to-mesenchymal transition (EMT/EndMT). Other suggested CAF precursors, although less common, include adipocytes^42^, pericytes^43^, mesothelial cells^44^, and smooth muscle cells^45^.

#### CAFs derived from recruited bone marrow cells

Although predominantly observed to be of local origin^38,46^, lineage tracing using murine models and human samples has revealed the potential of bone marrow contribution to the CAF pool in several neoplasias, including rectal adenoma^47^, gastric cancer^47^, hepatocellular carcinoma^48^, PDAC^49^, and breast cancer^50^. Mesenchymal stem cells (MSCs) recruited from the bone marrow can differentiate into a subpopulation of CAFs under tumor-derived TGF-β, WNT, and IL-6/STAT3 signaling^51,52^. Furthermore, the finding that BM-derived multilineage hematopoietic cells were engrafted in the tumors of recipient mouse models suggests the possibility that other bone marrow-derived cells may serve as CAF precursors. Indeed, some studies have shown that bone marrow macrophages/monocytes can convert into CAFs in PDAC^53,54^ and Lewis lung carcinoma (LLC)^55^ via macrophage–myofibroblast transition (MMT).

Collectively, overwhelming evidence now points toward multiple origins contributing to the CAF pool rather than one^28^. In fact, a collection of diverse subpopulations of CAFs from different progenitors coexist in distinct tumor types^56^ and coevolve with epithelial genetic events during the development of cancer, resulting in the temporal and spatial dynamics of CAFs^20^. Thus, it is tempting but also challenging to investigate the full CAF reservoir and the mechanisms governing the transformation from normal precursors to CAF subtypes during cancer evolution to gain an in-depth understanding of the tumor-associated stroma for targeted anticancer therapy.

### CAF heterogeneity and plasticity

While the presence of CAF subtypes based on their distinct expression patterns has long been accepted, functional categorization was first recapitulated in a coculture system of PDAC organoids and murine PSCs, which identified two mutually exclusive subtypes of CAFs^7^, termed αSMA^high^ IL-6^low^ myofibroblasts (myCAFs) and αSMA^low^ IL-6^high^ inflammatory CAFs (iCAFs). These two phenotypes, confirmed later in patient-derived PDAC specimens^9^, are dependent on their spatial location and biomedical niche within the TME. For example, myCAFs, activated by direct contact with neoplastic cells, reside adjacent to tumor foci, whereas iCAFs, induced by cancer cell-derived factors such as IL-1α and TNFα^7,57^, are located more distant from tumor cells. While iCAFs are generally confirmed to be tumor-promoting via the secretion of inflammatory cytokines and growth factors^58^, which confer proliferation, metastasis and chemoresistance of cancer cells^59^, myCAFs exhibit dual tumor-restraining and tumor-promoting roles, depending on the stage of the tumor and the complex context of the surrounding TME. One school of thought is that activation of fibroblasts reflects a host defense mechanism acting as a dense barrier limiting tumor spread^3^. It is also possible that the cues emanating from the TME may contribute to the opposing effects of myCAFs at different stages of tumor development^60^. Importantly, the distinct subsets of CAFs are not permanent but interconvertible via manipulation of specific signaling, as evidenced by conversion of iCAFs to myCAFs via the TGFβ signaling pathway, supporting the notion that CAF subpopulations have high potential for plasticity rather than terminally differentiated states^7^ and thereby providing a rationale for the induction of CAF phenotypic switching as a strategy in developing anticancer therapy.

Recently, a novel subpopulation characterized by the expression of major histocompatibility complex class II molecules was identified and termed antigen-presenting CAFs (apCAFs), suggesting an immunodulatory function of CAFs^8^. Indeed, the flow cytometry analysis of KPC tumors identified 3 distinct populations of CAFs with MHCII as a unique marker for apCAFs, while Ly6C as an iCAF-specific surface marker and myCAFs are the Ly6C^-^MHCII^-^ population^8^. Furthermore, the authors comprehensively evaluated and validated the transcriptomes of each subtype, providing novel marker genes for these cells^8^ (Table 1). In contrast to other CAF subtypes, apCAFs are considered to be immunosuppressive by inducing T regulatory (Treg) cell formation in breast^61^ and pancreatic tumors^62^, whereas recent work points to apCAF-participated T-cell immunity against lung tumors^63^, suggesting context-dependent tumor-promoting or tumor-suppressive effects of apCAFs.

Furthermore, broad intra- or intertumoral heterogeneity has been more evident by single-cell sequencing and multiomics approaches. New spatially and functionally distinct CAF subpopulations have been increasingly identified in different cancer types, including vascular CAFs (vCAFs), cycling CAFs (cCAFs), and developmental CAFs (dCAFs) in breast cancer, which emerge at different stages of tumor progression and thus play distinct roles in cancer development^20^. More importantly, based on scRNA-seq analysis of primary intrahepatic cholangiocarcinoma (ICC) tissues and orthotopic murine models, Aoki et al. identified a distinct CAF population that exhibited a higher response to treatment with anti-placental growth factor (PlGF). Blockade of PIGF resulted in an enrichment of a more quiescent subset with a reduced myofibroblast-like phenotype, suggesting that PIGF could be a key regulator of the CAF balance between quiescence and activation states in ICC^64^. More excitingly, Buechler et al. constructed single-cell and tissue atlases of fibroblast gene expression in health and disease and revealed the existence of a common lineage-wide fibroblast in all organs^65^. The stem-like ‘universal’ type of fibroblast cell, marked by expression of peptidase inhibitor 16 (Pi16) and Col15a, found in the steady state across tissues, serves as a reservoir that can yield specialized fibroblasts not only in normal tissues but also, more importantly, in the context of disease or injury where they undergo transition into highly activated fibroblasts, as observed by the development of LRRC15^+^ CAFs in PDAC.

Furthermore, the mechanism regulating the differentiation of universal Pi16^+^ fibroblasts into LRRC15^+^ CAFs has been unveiled recently. Krishnamurty et al. found that among 4 CAF clusters in a PDAC murine model, LRRC15^+^ CAFs, which were absent in normal tissues, emerged as the dominant CAF population under TGF*-*β signaling during tumor development and resulted in the suppression of antitumor immunity of cytotoxic T cells^66^. Interestingly, while Pi16 was demonstrated to be a marker of universal fibroblasts with stem-like features^65,66^, Elyada et al. previously suggested that Pi16 could be specific to iCAFs, as it showed higher expression in Ly6C^+^ cells, an iCAF marker identified in this study, than in other populations^8^. The controversy reflects the complexity and incomplete understanding of CAF subpopulations and suggests that the Pi16 subset could be an interesting population that needs further investigation.

In addition, based on cell-surface molecules, a subset of CD10^+^ GPR77^+^ CAFs has been defined, which was shown to correlate with chemoresistance by sustaining cancer stemness and may suffice as a prognostic indicator in breast and lung cancer^67^. Although various subsets of CAFs are emerging, further research is required to understand the full CAF pool in a cancer-dependent manner. A precise understanding of the mechanisms governing CAF heterogeneity and plasticity is a prerequisite for therapeutic interventions that selectively target tumor-supporting CAFs.

### The mechanisms regulating CAF heterogeneity

#### Genetic manipulation

**Fig. 2:**
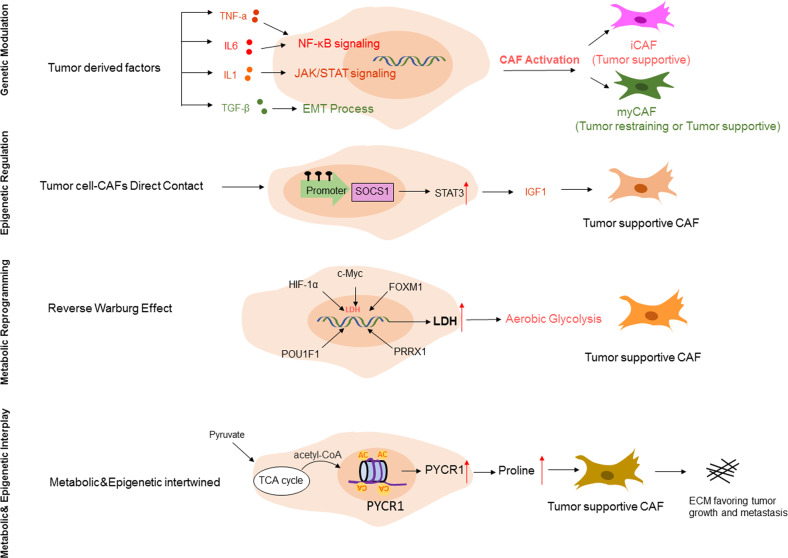
The molecular mechanisms regulating CAF heterogeneity. The diversity of CAF subtypes is regulated by complex molecular mechanisms, including genetic regulation mediated by tumor cell-derived factors, epigenetic modulation via direct contact between tumor cells and CAFs, and metabolic reprogramming reflecting the reverse Warburg effect. Moreover, these mechanisms can work independently or cooperatively to shape the stromal structure and function, resulting in tumor restraining or tumor supportive effects of CAF subsets in a context-dependent manner. SOCS1: Suppressor of cytokine signaling; STAT3: Signal transducer and activator of transcription 3; LDH: Lactate dehydrogenase; PYCR1: pyrroline-5-carboxylate reductase 1.

In general, CAF heterogeneity could arise from cancer cell-derived genetic evolution, epigenetic modulation or metabolic reprogramming (Fig. 2). Tumor cells educate CAFs, as evidenced by the difference in signature genes expressed by fibroblasts cocultured with different tumor cells. A successful education process can be manipulated by extrinsic or intrinsic factors. In particular, CAF plasticity can be induced by numerous tumor cell-derived growth factors and chemokines, including TGF*-*β, epidermal growth factor (EGF), PDGF, fibroblast growth factor (FGF), interleukin 6 (IL-6), and interleukin 1β (IL-1β)^68^, which skew CAFs toward specific subsets via activation of key regulatory pathways. For example, TGF*-*β can specify a myofibroblastic phenotype, while IL-6 particularly drives an immune-modulating phenotype in activated PSCs^69^. More importantly, the genetic heterogeneity of tumor cells profoundly and dynamically defines the CAF phenotype to fulfill their own growth need, highlighting the requirement of personalized medicine. For example, p53, one of the most commonly mutated genes in cancer, has been found to differentially shape the pancreatic stroma based on p53 status. Gain-of-function (GOF) mutant p53 induced a dominant population of CAFs with more permissive invasion and metastasis, while loss of p53 resulted in a more fibrotic stroma compared with wild-type p53 controls^70,71^. This p53-driven hierarchy in the PDAC stroma could be attributed to downstream paracrine signaling, such as TNF-a, nuclear factor κB (NF-κB)^71^, or exosomal secretion of certain cargo, such as podocalyxin (PODXL)^72^, indicating the influence of epithelial genetic events on CAF phenotype and behavior.

#### Epigenetic modulation

Although rarely harboring genetic aberrations, CAFs have been demonstrated to have highly consistent epigenome changes in the TME, as evidenced by genome-wide DNA methylation profiles of CAFs^73–75^. These differentially methylated regions were found to be particularly enriched at regulatory regions of the genome^74^ and key transcription factor-binding sites such as androgen receptor in prostate cancer^75^ and estrogen receptor (ER) in breast cancer^74^, resulting in local DNA hypermethylation and global DNA hypomethylation^76^. Alterations in the epigenetic landscape constitutively activate fibroblasts with specialized ECM remodeling capability^77^, robust autocrine signaling^69^ and dynamic immunomodulatory functions^77^. Furthermore, emerging evidence has revealed that the epigenetic switch induced by the crosstalk between cancer cells and CAFs can specifically reprogram the CAF subtype toward a proinvasive state. For example, it has been reported that normal stromal fibroblasts at baseline in the absence of epigenetic changes are naturally hostile to tumorigenesis. Direct contact of MSCs with PDAC cells triggered the induction of MSC DNA methylation in a global panel of genes, including SOCS1. Methylation of the SOCS1 promoter region led to its downregulated expression, resulting in the derepression of STAT3 signaling and subsequent release of procancerous growth factors such as insulin-like growth factor-1 (IGF-1). Moreover, it has been indicated that the tumor-supportive ability of CAFs is, at least in part, associated with the SOCS1 methylation status, as CAFs with SOCS1 methylation are stronger in promoting PDAC growth than CAFs without SOCS1 methylation, highlighting the importance of epigenetic modulation in shaping CAF functional heterogeneity^78^.

Since histone methylation is metabolically sensitive to cellular methylation potential^79^, epigenetic modulation often collaborates with metabolic reprogramming. Recent work identified nicotinamide N-methyltransferase (NNMT) as a master regulator sustaining the protumorigenic phenotype of CAFs via genome-wide DNA and histone hypomethylation. Mechanically, elevated expression of NNMT in the tumor stroma resulted in depletion of SAM (S-adenosyl methionine), a universal methyl donor for histones, which then reduced the global methylation potential of the cell, leading to upregulation of thousands of genes, including protumorigenic cytokines and oncogenic ECM components^79^. Similarly, in a recently published work, Kay et al. reported a mechanism driven by epigenetic and metabolic interplay in collagen-rich ECM production. Specifically, CAF proline is upregulated via hyperacetylated histone 3 at the promoter sites of pyrroline-5-carboxylate reductase 1 (PYCR1), a key enzyme for proline synthesis. The abundant proline provides CAFs with tumor collagen, resulting in the deposition of pro-tumorigenic extracellular matrix^80^.

In addition to primary tumors, CAF epigenetic heterogeneity has also been implicated in metastatic niches, where CAFs isolated from liver metastasis and lung metastasis of PDAC displayed distinct DNA methylation patterns, resulting in a more homogenous iCAF phenotype in liver metastasis, whereas CAFs from lung metastasis maintained heterogeneity^81^. The epigenetic shift toward a more homogeneous iCAF phenotype could, at least in part, explain the aggressive feature of liver metastasis and may provide the rationale for targeting the epigenome in PDAC liver metastasis.

#### Metabolic reprogramming

Since 1920, the “Warburg effect”, which proposes a model of cancer cell metabolic shift toward aerobic glycolysis, has been the leading principal in understanding the metabolic characteristics of malignant cells. Interestingly, recent investigations have found that the tumor stroma relies on the “reverse Warburg effect” to feed adjacent cancer cells where aerobic glycolysis occurs in CAFs and generates energy-rich metabolites such as lactate that tumor cells then use to perform oxidative phosphorylation^82^. Thus, metabolic adaptations are another feature of CAF activation toward the glycolytic phenotype and the acquisition of tumor-promoting functions. A number of possible mechanisms have been described to explain the metabolic switch of CAFs from oxidative phosphorylation to aerobic glycolysis. Lactate dehydrogenase (LDH), an enzyme involved in the conversion of pyruvate to lactate, is a key component of the glycolysis pathway, and various studies have therefore focused on the regulatory mechanism of fibroblast LDH expression in the context of tumors.

Intratumoral hypoxia is a typical hallmark of solid tumors, leading to the stabilization of HIF-1α. Sustained elevation of HIF-1α directly induces glycolytic enzymes such as LDH and pyruvate kinase M2 (PKM2), thereby altering CAF metabolism toward a pro-glycolytic phenotype^83,84^. In addition to HIF-1a, other factors have been shown to directly regulate the transcription of glycolytic enzymes, including oncogenic c-Myc^85^ and forkhead box protein M1 (FOXM1)^86^, along with the recently identified POU1F1^87^ and PRRX1 (paired related homeobox1)^88^. Moreover, chronic hypoxia can lead to hypomethylation of glycolysis-related genes in fibroblasts, suggesting that metabolic coupling with epigenetic events favors the accumulation of pro-glycolytic CAFs. Indeed, chromatin modifiers and transcription factors have been found to act on CAF expression of glycolytic enzymes in a cooperative manner^89^.

Crosstalk between cancer cells and the TME can also influence the plasticity of stromal cells, as evidenced by the observation that cancer cells with high metastatic potential have a higher capability to metabolically reprogram fibroblasts, which show a more aggressive phenotype, than fibroblasts reprogrammed by low metastatic cancer cells^90^, supporting the notion that CAFs are the metabolic scar of cancer and represent an important target as cancer cells per se in anticancer therapy.

## Development of novel therapeutic strategies against tumor-promoting CAFs

**Fig. 3:**
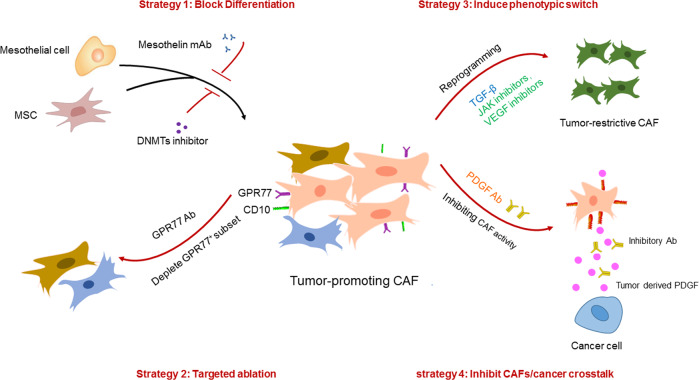
Therapeutic strategies of stroma re-engineering. Current strategies to selectively target tumor-promoting CAFs include (**a**) blocking the differentiation from precursor cells to tumor-promoting CAFs via inhibition of precursor activation or targeting key signaling pathways in differentiation. **b** Targeted depletion of tumor-promoting CAFs without affecting tumor-restraining CAFs through genetic manipulation or specific antibodies. **c** Induction of phenotypic switching from tumor-promoting to tumor-restraining CAFs. **d** Targeting the crosstalk between cancer cells and tumor-promoting CAFs to inhibit their supportive effect on cancer proliferation, migration and chemoresistance.

Over the past decade, multiple approaches have attempted to target CAFs and their crosstalk with cancer cells in preclinical models and clinical trials. Direct depletion of CAFs through genetic manipulation, pharmacological targeting or specific antibodies, contrary to previous assumptions, enhanced tumor growth and aggressiveness following ablation of α-SMA^+^ CAFs in PDAC^6^. Depletion of FAP^+^ CAFs, although has been reported to prolong survival in PDAC murine models^91^, administration of sibrotuzumab, a FAP-specific antibody, failed to improve survival for patients with metastatic colorectal cancer in a Phase II trial^92^, highlighting the importance of targeted therapy rather than widespread eradication of all CAFs. To date, novel approaches specifically targeting tumor-supportive CAFs and re-engineering the tumor stroma have been explored extensively in preclinical models, some of which have begun to enter clinical trials (Fig. 3).

### Inhibition of progenitor cell differentiation toward a pro-cancerous CAF

To date, elucidation of the molecular mechanisms underlying CAF subset formation has made it possible to inhibit the transition from precursor cells toward tumor-promoting CAFs. It has been reported that during pancreatic cancer progression, tumor paracrine signals, including IL-1 and TGF-β, induce the mesothelial to apCAF transition, which is responsible for immunosuppression^62^. However, IL-1 and TGF-β may not serve as good targets for specific inhibition of the transition due to their pleiotropic effects. Therefore, a monoclonal antibody (mAb) against mesothelin (MSLN), a specific mesothelial cell marker, was tested and found to effectively block mesothelial cell activation and apCAF generation^62^.

Epigenetic reprogramming has been revealed to provide dynamic and reversible modulation of stromal cells. Therefore, targeting the epigenome via regulation of DNA methylation or histone modification has been investigated in preclinical models, and several promising molecules are now active in clinical trials. Decitabine (DAC), a DNA methyltransferase (DNMT) inhibitor, was first applied in the treatment of a PDAC xenograft model in which the tumor-free survival with DAC-pretreated MSCs was significantly longer than that of mice with untreated MSCs^78^. However, it is notable that inhibition of DNA methylation only attenuated but could not fully prevent MSC reprogramming into protumorigenic CAFs, suggesting that multiple mechanisms cooperate in driving CAF differentiation. Following this, NNMT-mediated histone methylation was reported to be a master regulator in defining the protumorigenic stroma. Strikingly, inhibition of NNMT activity led to a reversion of the CAF phenotype in orthotopic high-grade serous ovarian cancer (HGSC), resembling normal omental fibroblasts morphologically and transcriptionally^79^. This finding implicates that the metastatic stroma can be targeted and even revert back to the normalized state via epigenetic remodeling, is of high translational value and should be further explored as a new therapeutic approach for advanced cancer.

### Targeted ablation of tumor-promoting CAFs

Selective targeting of tumor-promoting CAFs relies on the identification of specific and convenient markers. It is proposed that CD10 and GPR77 can define a human CAF subset that sustains cancer stemness and chemoresistance. Depletion of the CD10^+^GPR77^+^ subset abrogated tumor growth and reversed chemosensitivity, and the effect also replicated with administration of GPR77 neutralizing mAb in a patient-derived xenograft model of breast cancer. Mechanistically, GPR77 not only serves as a surface marker but also functions as an essential signaling molecule for the maintenance of NF-κB activities in the subset^67^.

Chemotherapy resistance in PDAC is often associated with desmoplasia^93,94^, one of which was recently shown to occur through PlGF/VEGF-mediated activation of CAFs in an orthotopic PDAC mouse model treated with gemcitabine^95^. Therefore, the authors delicately designed and synthesized a multiparatopic VEGF decoy receptor (Ate-Grab), a fusion protein consisting of single-chain Fv of atezolizumab for targeting PD-L1-rich tumor tissues, fused to VEGF-Grab, which was previously developed to target PIGF/VEGF^96^, resulting in PD-L1-directed PlGF/VEGF blockade. Indeed, Ate-Grab treatment relieved chemotherapy-induced desmoplasia in a PDAC model by sequestering PlGF/VEGF within the TME and thus inhibiting CAF activation, especially the CD141^+^ population, a key subset activated by gemcitabine treatment responsible for tumor fibrosis^95^.

However, specific markers to precisely define a unique human CAF subpopulation in vivo are still lacking in many tumor types; therefore, targeting the potential cellular sources of CAFs may provide another way for precision treatment. PSCs are known to give rise to PDAC CAFs, although recent studies have demonstrated multiple cells of origin in addition to PSCs. To functionally dissect the role of PSC-derived CAFs in PDAC progression, PSC CAFs were specifically ablated via Cre-mediated diphtheria toxin (DT) treatment, which resulted in attenuated tumor stiffness and a desmoplastic milieu with lower levels of matricellular proteins^36^. This finding is worthwhile for further investigation since PDAC patients with abundant matricellular fibrosis have been observed to have shortened survival^97^. Similarly, in the liver, HSCs, as the main source of CAFs, have been targeted using the same strategy, leading to a reduced tumor burden in ICC^60^. Notably, HSCs were found to give rise to both myCAFs and iCAFs in this study; therefore, targeted ablation of HSCs not only blocks iCAF but also myCAF formation. Further analysis demonstrated that myCAFs in ICCs act as tumor promoters via the interaction of hyaluronan (HA) receptors and hyaluronan synthase 2 (Has2)^60^, highlighting a context-dependent myCAF function and the necessity of a thorough understanding of CAF subset function in vivo.

### Induction of phenotypic switching toward tumor-restrictive CAFs

Compared with targeted ablation of CAF subsets, fine-tuning of specific CAF subpopulations based on their inherent plasticity seems to be more feasible and less challenging for therapeutic interventions. Mounting evidence has pointed to TGF-β1 signaling as one of the key regulators governing the fate of CAF subpopulations, especially between myCAF and iCAF conversion in PDAC, which is under preclinical investigation by transforming tumor-promoting iCAFs into tumor-restrictive myCAFs through activation of TGF-β signaling^69^. In addition to the myCAF and iCAF transition, TGF-β signaling has also been found to regulate CAF functional heterogeneity in other tumors. In a recent study, Hu et al. established a living biobank of CAFs from non-small lung cancer (NSCLC) patients, which displayed distinctive functional subsets with subtypes I and II as cancer cell protectors, while subtype III correlated with higher sensitivity to chemotherapy. Furthermore, these subsets were interconvertible by intrinsic TGF-β1 signaling, where loss of TGF-β signaling increased tumor-protection subsets, whereas addition of TGF-β1 downregulated these subsets and thereby attenuated their capacity to confer EGFRi resistance^98^.

Intrinsic or acquired trastuzumab resistance has been found in some HER2^+^ breast cancer patients and results in the failure of standard trastuzumab therapy^99^. Recently, it was demonstrated that the abundance of CD16^+^ fibroblasts in HER2^+^ breast cancer patients is correlated with poor response to trastuzumab through severe desmoplasia and inefficient drug delivery. Mechanistically, activated CD16+ fibroblasts enhanced matrix production and stiffness through a VAV2-dependent pathway^100^. Targeting VAV2 not only blocked the activation of CD16+ fibroblasts but also reversed desmoplasia and decreased trastuzumab resistance^100^.

The discovery of the IL1α/JAK/STAT signaling pathway in promoting iCAF formation has provided novel pharmacological targets. JAK inhibitors are reported to skew the iCAF subtype toward the myCAF subtype in PDAC^69^. Furthermore, preclinical studies have confirmed the effectiveness of the IL-1 receptor antagonist anakinra on PDAC progression^101^, which has now entered a phase 1 clinical trial in combination with standard chemotherapy in PDAC (NCT02021422). Indeed, a series of clinical trials targeting the specific pathway essential for procancerous CAF formation or maintenance, including TGF-β, VEGF and FGF, have shown promising antitumor efficacy and acceptable safety in combination with chemotherapy^102–106^ (Table 2). However, CAF-targeted clinical trials may not always recapitulate the advantageous effect in preclinical models, as observed by the diminished survival of pancreatic cancer patients treated with the hedgehog inhibitor IP-926 (NCT01130142) or vismodegib (NCT01383538) in combination with gemcitabine. The disappointing outcomes highlight the importance of a careful, thorough and long-term preclinical investigation before translating optimism in the CAF field into the clinic.

**Table 2:** Completed and active clinical trials targeting CAFs.

Target/Mechanism	Drug	Combination Therapy	Phase	Cancer type	Clinical Trial Outcome	Clinical Trial NO
TGF-βR inhibitor	Galunisertib	Gemcitabine (nucleoside analog)	Ib/II	Metastatic unresectable pancreatic cancer	Prolonged OS, with minimal added toxicity	NCT01373164
	Galunisertib	Durvalumab (PD-L1) antibody)	Ib	Metastatic pancreatic cancer	Acceptable tolerability and safety profile	NCT02734160
	Galunisertib	Sorafenib (multikinase inhibitor) or Ramucirumab (VEGFR-2 mAb)	II	Advanced hepatocellular carcinoma	Prolonged OS, with acceptable safety	NCT01246986
Anti-PD-L1/TGF-βR fusion protein	SHR-1701	N/A	I	Metastatic or locally advanced solid tumors	Showed encouraging antitumor activity and controllable safety^102^	NCT03774979
	M7824	N/A	I	Metastatic or locally advanced solid tumors	Showed a manageable safety profile^103^	NCT02517398
Immunocytokine consisting of IL-2 variant targeting FAP-α	RO6874281	Trastuzumab (HER-2 mAb) or Cetuximab (EGFR mAb)	I	Breast cancer Cancer of head and neck	No Results Posted	NCT02627274
	RO6874281	Pembrolizumab (PD-L1 antibody)	I	Metastatic melanoma	No Results Posted	NCT03875079
	RO6874281	Atezolizumab (PD-L1 antibody) or Gemcitabine (nucleoside analog) or Vinorelbine (anti-mitotic agent)	II	Advanced/metastatic head and neck oesophageal and cervical cancers	No Results Posted	NCT03386721
mAb IL-6	CNTO 328 (siltuximab)	N/A	II	Hormone refractory prostate cancer	Showed biologic activity but minimal clinical activity as monotherapy. Combination with chemotherapy studies are ongoing^106^	NCT00433446
VEGFR inhibitor	Lenvatinib	Ifosfamide (DNA alkylator) or Etoposide (topoisomerase II inhibitors)	I / II	Solid malignant tumors Osteosarcoma Differentiated Thyroid Cancer	Demonstrated the safety and preliminary antitumor activity of lenvatinib^104^	NCT02432274
FGFR inhibitor	Pemigatinib	N/A	II	advanced/metastatic or surgically unresectable cholangiocarcinoma	PFS advantage of pemigatinib versus other systemic therapies in patients with FGFR2 fusions/rearrangements^105^	NCT02924376
IL-1 inhibitor	Anakinra	Oxaliplatin or Irinotecan or fluorouracil (standard chemotherapy)	I	Metastatic PDAC	No Results Posted	NCT02021422
PDGFR inhibitor	CP-868,596	Docetaxel (anti-mitotic agent) or AG-013736 (VEGFR Inhibitor)	I	Advanced Solid Tumors	No Results Posted	NCT00949624

*OS* overall survival, *PFS* progression-free survival, *mAb* monoclonal antibody, *TGF-βR* transforming growth factor-β receptor, *FAP* fibroblast activation protein, *VEGFR* vascular endothelial growth factor receptor, *FGFR* fibroblast growth factor receptor, *IL-2* interleukin-2, *PDGFR* platelet-derived growth factor receptor.

### Inhibiting activity of mature CAFs

Due to the existence of high intra- and intertumor heterogeneity as well as a lack of CAF-specific markers, researchers are more interested in elucidating the crosstalk between cancer cells and CAFs, especially how CAFs foster excessive proliferation and chemoresistance, to inhibit the tumor-promoting activity of CAFs by disrupting signaling pathways. It has been found that paracrine communication between cancer cells expressing PDGF ligands and CAFs expressing cognate receptors leads to the specification of basal-like breast tumors, a hormone receptor-negative subtype that cannot benefit from tamoxifen treatment in the clinic^107^. Surprisingly, neutralization of the PDGF pathway with administration of an inhibitory antibody resulted in conversion from the basal-like subtype into the luminal subtype, which is susceptible to anti-estrogen therapies^107^. In addition, it has been reported that pancreatic tumors harboring p53 mutations promote CAF reprogramming toward a prometastatic subset with the secretion of heparin sulfate proteoglycan 2 (HSPG2) in the stroma. HSPG2 deposition creates a permissive environment for invasion and metastasis, and depletion of HSPG2 not only impairs metastasis but also improves chemotherapy efficacy in pancreatic tumors harboring a GOF p53 mutation^71^, providing a potential avenue toward targeting the stromal feedback of aggressive subsets rather than manipulation of the CAF phenotype.

## Conclusions

Although CAFs have long been investigated as a crucial player in cancer development and therefore represent an attractive therapeutic target, clinical trials targeting CAFs have mostly ended in failure and even in some cases, accelerated cancer progression^1^, demonstrating that the dynamic complexity of CAF identity and function is far beyond our current understanding. Therefore, dissecting the heterogeneous subpopulations and diverse functions of CAFs in a context-dependent manner is of high importance.

Single-cell analysis techniques such as single-cell transcriptomic and proteomic technologies provide a powerful tool for deciphering cellular heterogeneity and identifying new precise biomarkers for targeted therapy. More importantly, functional assessment of each subset regarding their crosstalk not only with cancer cells but also with other stromal components and even between distinct CAF subpopulations should be given sufficient attention to classify CAF subsets into functional categories. Moreover, given the diversity of CAF reservoirs and the plasticity of CAF changes over time with tumor progression, it would be interesting to illustrate the relationship between each CAF subpopulation, whether they are hierarchical, parallel, or overlapping. For this purpose, reliable in vivo tumor models combined with genetic manipulation systems such as the Cre–lox system offer an elegant way to interrogate CAF function at different stages of tumor development.

While current studies focus on the identification of novel tumor-promoting CAF subsets and strategies to target them specifically, it is noteworthy that tumor-suppressive CAF populations and their homeostatic maintenance will also be worthwhile to identify, as enhancement of these functions as a barrier restricting tumor spread or reprogramming of the so-called bad CAF into good CAF should be a goal of future stroma-targeted therapies. Therefore, there is a growing appreciation that therapeutic approaches to normalize or re-engineer the tumor stroma into a quiescent state or even tumor-suppressive phenotypes would offer the potential for translational impact in improving patient survival.
